# Skeletal muscle proteomics: current approaches, technical challenges and emerging techniques

**DOI:** 10.1186/2044-5040-1-6

**Published:** 2011-02-01

**Authors:** Kay Ohlendieck

**Affiliations:** 1 Muscle Biology Laboratory, Department of Biology, National University of Ireland, Maynooth, County Kildare, Ireland

## Abstract

**Background:**

Skeletal muscle fibres represent one of the most abundant cell types in mammals. Their highly specialised contractile and metabolic functions depend on a large number of membrane-associated proteins with very high molecular masses, proteins with extensive posttranslational modifications and components that exist in highly complex supramolecular structures. This makes it extremely difficult to perform conventional biochemical studies of potential changes in protein clusters during physiological adaptations or pathological processes.

**Results:**

Skeletal muscle proteomics attempts to establish the global identification and biochemical characterisation of all members of the muscle-associated protein complement. A considerable number of proteomic studies have employed large-scale separation techniques, such as high-resolution two-dimensional gel electrophoresis or liquid chromatography, and combined them with mass spectrometry as the method of choice for high-throughput protein identification. Muscle proteomics has been applied to the comprehensive biochemical profiling of developing, maturing and aging muscle, as well as the analysis of contractile tissues undergoing physiological adaptations seen in disuse atrophy, physical exercise and chronic muscle transformation. Biomedical investigations into proteome-wide alterations in skeletal muscle tissues were also used to establish novel biomarker signatures of neuromuscular disorders. Importantly, mass spectrometric studies have confirmed the enormous complexity of posttranslational modifications in skeletal muscle proteins.

**Conclusions:**

This review critically examines the scientific impact of modern muscle proteomics and discusses its successful application for a better understanding of muscle biology, but also outlines its technical limitations and emerging techniques to establish new biomarker candidates.

## Introduction

Proteomics is an unbiased and technology-driven approach for the comprehensive cataloging of entire protein complements and represents an ideal analytical tool for the high-throughput discovery of protein alterations in health and disease [1]. Mass spectrometry-based proteomics is concerned with the global analysis of protein composition, posttranslational modifications and the dynamic nature of expression levels [2-4]. The generation of large data sets on protein expression levels makes proteomics a preeminent hypothesis-generating approach in modern biology [5]. Proteomics has now been accepted as a key technology in biochemistry, cell biology, systems biology and drug discovery [6-9]. In this respect, proteomics suggests itself as a thorough approach for the detailed biochemical analysis of heterogeneous and plastic types of tissue, such as muscles. Skeletal muscle proteomics aims at the global identification, detailed cataloguing and biochemical characterisation of the entire protein complement of voluntary contractile tissues in normal and pathological specimens [10-12]. Although mass spectrometry-based proteomics is a relatively new analytical approach in the general field of muscle biology, large-scale proteomic studies have already provided a plethora of new information on global changes during myogenesis, fibre maturation, muscle transformation and natural muscle aging [12-14]. High-throughput surveys of common neuromuscular diseases, such as x-linked muscular dystrophy [15], have revealed many new proteome-wide changes and adaptations on the molecular and cellular level [13]. Proteomics routinely employs high-resolution separation techniques, such as two-dimensional gel electrophoresis and/or liquid chromatography, in combination with advanced mass spectrometric methods for the unequivocal identification of peptides and proteins of interest [16-19]. The independent verification of proteomic data is usually accomplished by using immunoblotting surveys, activity assays and immunofluorescence microscopic analysis [12]. Over the past few years, technical advances in mass spectrometry [20-22] and the development of vastly improved bioinformatic analysis tools [23-25] have driven the remarkable progress of proteomic science. This review outlines the findings from recent applications of mass spectrometry-based proteomics for studying physiological adaptations and pathological alterations in skeletal muscle tissues and critically examines novel analytical strategies to establish muscle-specific biomarker signatures.

### The complex biochemistry of skeletal muscle tissues

Contractile fibres of skeletal muscle tissues constitute the cellular units that provide coordinated excitation-contraction-relaxation cycles for voluntary movements and postural control [26]. In addition, skeletal muscles play a central physiological role in heat homeostasis and present a crucial metabolic tissue that integrates various biochemical pathways. For example, skeletal muscle fibres have the highest capacity for insulin-mediated uptake of glucose in the body, making muscle tissues a critical organ in carbohydrate metabolism [27]. The complex cellular tasks of muscles are performed by a large number of proteins with specialised functions, structures and interactions. Skeletal muscles contain a considerable amount of integral membrane proteins and high molecular mass complexes. Some of the largest protein species present in mammalian tissues are expressed in skeletal muscle, such as nebulin of 600-800 kDa and titin with a molecular mass exceeding 1,200 kDa [28,29]. Although supramolecular membrane assemblies are also found in many other types of tissue, notably the nervous system, they represent a major biochemical feature of contractile fibres. This includes the nicotinic acetylcholine receptor of the postsynaptic muscle membrane [30], the acetylcholinesterase of the basal lamina [31], the voltage-sensing dihydropyridine receptor of the transverse tubules [32], the ryanodine receptor Ca^2+^-release channel of the triad junctions [33], the dystrophin-glycoprotein complex of the sarcolemma [34], the respiratory chain of muscle mitochondria [35] and the abundant actomyosin machinery with its regulatory troponin-tropomyosin system [36]. The large number of membrane-associated proteins, the exceptionally high molecular mass of many muscle components, extensive posttranslational modifications in various muscle proteins and their organisation in highly complex supramolecular structures make it extremely difficult to carry out conventional biochemical studies of potential changes in protein clusters during physiological adaptations or pathological processes. In this respect, proteomics and subcellular proteomics attempt to isolate, separate and identify the entire protein constellation of a given muscle tissue or fibre population. Most initial proteomic studies have focused on total extracts of mostly soluble muscle proteins, and more recent investigations have made an effort to also encompass integral proteins and high molecular mass components in their analysis.

### Dynamics and heterogeneity of the skeletal muscle proteome

**Figure 1:**
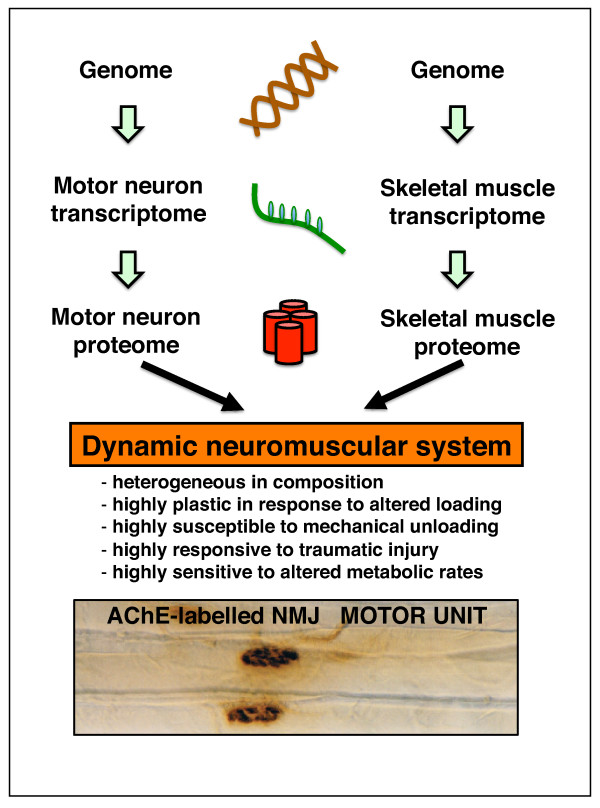
**Biological hierarchy of the neuromuscular system**. Shown are the organisation of the genome, transcriptome and proteome of motor neurons and skeletal muscles. The histological image illustrates neuromuscular junctions on individual muscle fibres labelled for the presence of the enzyme acetylcholinesterase.

The biological hierarchy from genome to transcriptome to proteome is diagrammatically shown in Figure 1. In contrast to the stable neuromuscular genome, the muscle transcriptome is highly dynamic and the muscle proteome is constantly changing and adapting to altered functional demands, making its comprehensive analysis very challenging. The estimated 25,000 protein-coding genes in the human genome probably translate into several hundred thousand different protein species [37]. Although alternative splicing and posttranslational modifications depend on the exact definition of the term 'protein isoform', they result in the production of a very large number of distinct subspecies of proteins. In mammals, the majority of genes appear to be differentially spliced, so that a single gene often codes for multiple proteins, which in turn may undergo different types of posttranslational modifications. A small number of muscle-specific genes can thus generate a large number of protein species encoded by the muscle genome. An excellent example of this phenomenon is the family of sarcoendoplasmic reticulum Ca^2+^-ATPase (SERCA)-type Ca^2+ ^pumps from slow and fast muscles. Although the SERCA proteins are encoded by three genes, of which the *SERCA1 *and *SERCA2 *genes are expressed in voluntary fibres, the isoform diversity of this abundant ion pump is drastically increased by alternative splicing of the transcripts and various posttranslational modifications [38], producing more than 10 different SERCA isoforms [39]. These cellular processes significantly increase muscle protein diversity.

The wide and dynamic expression range of proteins within a specific tissue makes it impossible to separate and detect all protein species with currently available biochemical techniques. In addition, most tissue types are heterogeneous in composition. Aside from the main contractile cells of differing contractile properties, such as slow oxidative (type I), moderately fast oxidative glycolytic (type IIa) and fast glycolytic (type IIb) fibres, muscle contains extended layers of connective tissues, capillaries and nerve cells [40-42]. Thus the starting material for almost all invasive biochemical studies, that is, homogenised muscle tissues, contains a certain degree of cell types that have originated from the tendon, epimysium, endomysium, perimysium, muscle spindles, satellite cells, blood vessels and motor neurons. This analytical fact has to be taken into account when one interprets proteomic findings from total tissue extracts. In the past, microassay systems have been developed to study single-fibre preparations biochemically [43], so it might be feasible to investigate such cellular preparations by using miniaturised separation protocols and proteomic techniques with enhanced sensitivity in the future. Concentration differences between highly abundant muscle proteins, such as glycolytic enzymes, and low-abundance muscle proteins, such as surface signaling receptors, have been estimated to be several orders of magnitude. Standard gel electrophoretic or liquid chromatographic methods are not capable of separating this vast range of muscle proteins with differing densities.

### Technical limitations of two-dimensional gel electrophoresis

Besides the biological fact that it is extremely difficult to accurately define a fixed protein complement in highly adaptable and dynamic fibre populations as 'the skeletal muscle proteome', the most obvious technical obstacle to the study of entire muscle proteomes is the limited availability of all-encompassing protein analytical capabilities. Currently no one set of biochemical techniques exists that can efficiently separate and consistently detect the total protein complement of a given cell type. Hence, with respect to interpreting findings from analytical gel electrophoresis, it is important to take into account various technical limitations. Two-dimensional gel electrophoresis of crude tissue extracts usually underestimates the presence of low abundance elements, the amount of integral membrane proteins and components with very high molecular masses in complex tissues [19,44-46]. In addition, proteins with extreme p*I *values are often not properly resolved at the edge of large gel systems with a wide p*I *range. This problem can be partially addressed by using narrow-range p*I *gels in the first dimension or by employing overlapping gel systems covering several p*I *ranges [44]. Another important issue in two-dimensional gel electrophoresis is the distortion of protein spots due to abundant protein species or the presence of isforms with extensive posttranslational modifications [45]. For example, high levels of heterogeneous glycosylation patterns can result in broad or overlapping spot patterns, which are difficult to pick for in-gel digestion procedures. Importantly, the presence of muscle proteins with a high density, such as myosin heavy chains, myosin light chains, troponins, tropomyosins and actins, can distort certain zones within the two-dimensional separation pattern and thus potentially contaminate other protein spots. In such a case, the densitometric analysis of a specific spot and the identification of the most abundant protein species present in this gel region might not perfectly correlate. Proteolytic degradation products of high molecular mass proteins may also complicate the analysis of the gel image.

However, despite these technical limitations, gel electrophoresis-based proteomics results in excellent coverage of soluble and abundant muscle proteins involved in the regulation and execution of the contraction-relaxation cycle, energy metabolism and the cellular stress response [13]. The use of crude tissue extracts as starting material has the advantage of representing the entire soluble protein complement without the potential danger of protein desorption or artefactual entrapment by complex separation steps. On the other hand, organelle and membrane proteomics reduces sample complexity by focusing on distinct subsets of protein populations, as outlined below. Ideally, proteomic studies of skeletal muscle tissues should first use both crude extracts and distinct subcellular fractions as starting material and second employ gel electrophoresis and liquid chromatography in parallel for the separation of as many different classes of muscle proteins as possible.

### Mass spectrometry-based muscle proteomics

**Figure 2:**
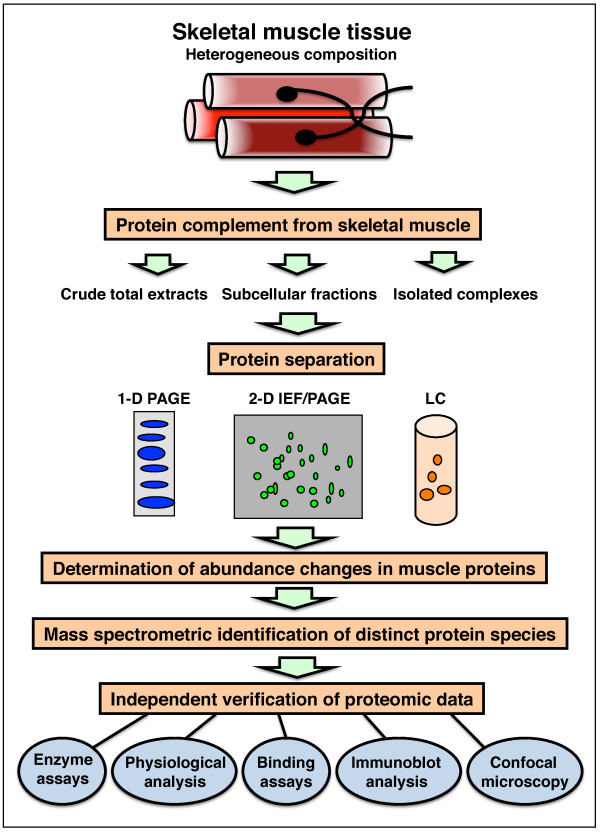
**Proteomic profiling of skeletal muscle**. The flowchart outlines the various preparative and analytical steps involved in the routine mass spectrometry-based proteomic investigation of contractile tissues. Protein separation is usually carried out by one-dimensional polyacrylamide gel electrophoresis (1-D PAGE), 2-D gel electrophoresis with isoelectric focusing in the first dimension and PAGE in the second slab gel dimension (2-D IEF-PAGE) and/or liquid chromatography.

The long-term goal of proteomic profiling studies is the cataloguing of all expressed protein species and the establishment of comprehensive biomarker signatures that characterise physiological processes during development and natural aging, as well as disease progression in common pathologies. The flowchart in Figure 2 outlines the main steps of proteomic profiling studies. The various steps involved in routine muscle proteomics involve (1) the extraction of the target protein complement, resulting in total crude preparations of mostly soluble components, distinct subcellular fractions including membrane-associated elements or isolated complexes; (2) the separation of proteins by one-dimensional gel electrophoresis, high-resolution two-dimensional gel electrophoresis and/or liquid chromatography; (3) in the case of gel electrophoretic approaches, the determination of altered expression levels in protein maps by densitometric surveys; (4) the mass spectrometric identification of distinct protein species, usually by analysing trypsin-generated peptide mixtures of proteins of interest; and (5) the validation of proteomic data by routine assay systems, such as immunoblotting, enzyme assays, confocal microscopy, binding assays and physiological tests.

Over the past few years, mass spectrometry-based proteomics has successfully catalogued several hundred of the most abundant and soluble muscle-associated protein species and identified several thousand distinct protein isoforms present in skeletal muscle tissues [47-50]. Muscle proteomics has been applied to the comprehensive biochemical profiling of developing, maturing and aging muscle [51-56], as well as the analysis of contractile tissues undergoing physiological adaptations seen in disuse atrophy, physical exercise and chronic muscle transformation [57-63]. Biomedical investigations into proteome-wide alterations in skeletal muscle tissues were also used to establish novel biomarker signatures of neuromuscular pathologies. Disease-specific markers were determined for muscle-associated diseases such as dystrophinopathy [64-66], dysferlinopathy [67], traumatic denervation [68], obesity [69], diabetes-related contractile weakness [70], sepsis [71], hypokalemic myopathy [72], inclusion body myositis [73] and reducing body myopathy [74]. Since skeletal muscle biology is highly relevant to the meat industry, muscle proteomics has been widely applied to cataloguing and studying protein complements in livestock [75-77]. These studies have especially focused on the proteomic evaluation of hypertrophy in chicken [78,79], sheep [80], pig [81-83] and cow [84] muscles. Table 1 lists key findings from recent proteomic studies focusing on the biochemical characterisation of skeletal muscle tissues. Biomarker signatures are listed only when more than one study has been conducted on a specific cell biological, physiological or pathological topic.

**Table 1:** List of select biochemical studies that have focused on the proteomic profiling of developing, transforming, pathological and aging skeletal muscle tissues

Proteomic study	Identification of muscle-specific biomarker signatures	References
Skeletal muscle protein complement	Profiling of the skeletal muscle-associated proteome from various species. Shotgun proteomics has catalogued more than 2,000 human skeletal muscle proteins.	[47-50]
Muscle development	Proteomic analysis of myogenesis has identified a large variety of proteins, including metabolic enzymes (enolase, aldehyde dehydrogenase), contractile and structural elements (myosins, actins, tubulin, desmin), stress proteins (peroxiredoxin, superoxide dismutase, heat shock proteins) and components involved in protein synthesis (ribosomal enzymes).	[51-54,136,137]
Muscle transitions	Proteomic studies have established distinct changes in marker characteristic of fast-to-slow muscle transformations: fatty acid-binding protein, albumin, myosin heavy chains, myosin light chains, tropomyosins, troponins, creatine kinase and myoglobin.	[62,63]
Effect of exercise	Proteomic profiling of physical training showed alterations in enolase, albumin, succinate dehydrogenase, myoglobin, aconitase and transferrin.	[60,61]
Muscle growth	Proteomic analysis of hypertrophy revealed considerable changes in the abundance of contractile proteins (troponins, myosins), metabolic proteins (fatty acid-binding protein, phosphoglucomutase) and various molecular chaperones.	[78-84]
Disuse atrophy	Proteomic profiling of muscle unloading showed drastic changes in structural and contractile proteins (myosins, actins, troponins), stress proteins (various heat shock proteins) and marker enzymes of slow-to-fast transitions (enolase, triosephosphate isomerase, lactate dehydrogenase, isocitrate dehydrogenase).	[57-59,135]
Dystrophinopathy	Proteomic screening of the x-linked muscular dystrophy (mdx) animal model of Duchenne muscular dystrophy revealed that the deficiency in dystrophin is associated with altered levels of metabolic enzymes (adenylate kinase, carbonic anhydrase, isocitrate dehydrogenase), Ca^2+^-regulatory proteins (regucalcin, calsequestrin) and molecular chaperones (cardiovascular heat shock protein cvHsp).	[64-66]
Muscle aging	Proteomic profiling of aging muscle tissues has shown changes in metabolic markers that are characteristic of a fast-to-slow transition process (various glycolytic enzymes, such as pyruvate kinase and numerous mitochondrial enzymes), as well as changes in adenylate kinase and various molecular chaperones.	[55,56,87,88,90,92,116,118]

Besides bulk skeletal muscle, refined studies of muscle subtypes with an unusual histology such as extraocular muscles have been conducted [66,85], including the proteomic profiling of sarcomere-associated elements [86]. In addition, posttranslational changes were studied in skeletal muscle preparations by proteomics, focusing especially on protein nitration [87], carbonylation [88], glycosylation [89-91] and phosphorylation [92,93]. Comprehensive reviews have covered the critical examination of these proteomic studies [10-15,94,95]. Thus, instead of recapitulating the considerable impact of these early studies of muscle proteomics, this review instead outlines the more recent application of fluorescent gel electrophoresis, organelle proteomics and membrane proteomics for studying skeletal muscle tissues.

### Fluorescence gel electrophoresis of the muscle proteome

Labeling of proteins with fluorescent dyes has been extensively applied in proteomic investigations [96-99]. However, the one technique that stands out for its potential to directly compare two different sets of protein complements is fluorescence difference in-gel electrophoresis, usually abbreviated as DIGE [100]. This advanced gel electrophoretic method represents one of the most powerful analytical tools for conducting comparative protein biochemical investigations [101]. The fluorescence DIGE technique covers the same type of proteins as conventional two-dimensional gel electrophoretic approaches. If proper labeling protocols are followed, the fluorescence tagging procedure does not significantly interfere with the chemical properties of proteins with respect to their gel electrophoretic mobility. Minden *et al*. [102] first described this two-dimensional gel electrophoretic technique and the first extensive evaluation of its two-dimensional software analysis was conducted by Tonge *et al*. [103]. The DIGE technique is an ideal method for comparing entire soluble proteomes in one swift analytical approach [100] if one accepts that proteins with extreme p*I *values, proteins with a very low density, certain classes of high molecular mass proteins, extremely hydrophobic proteins and elements with certain posttranslational modifications may be underrepresented in two-dimensional gel systems [19,44-46]. DIGE greatly reduces gel-to-gel variations and thereby greatly improves the evaluation of trends in changed protein expression patterns [104].

**Figure 3:**
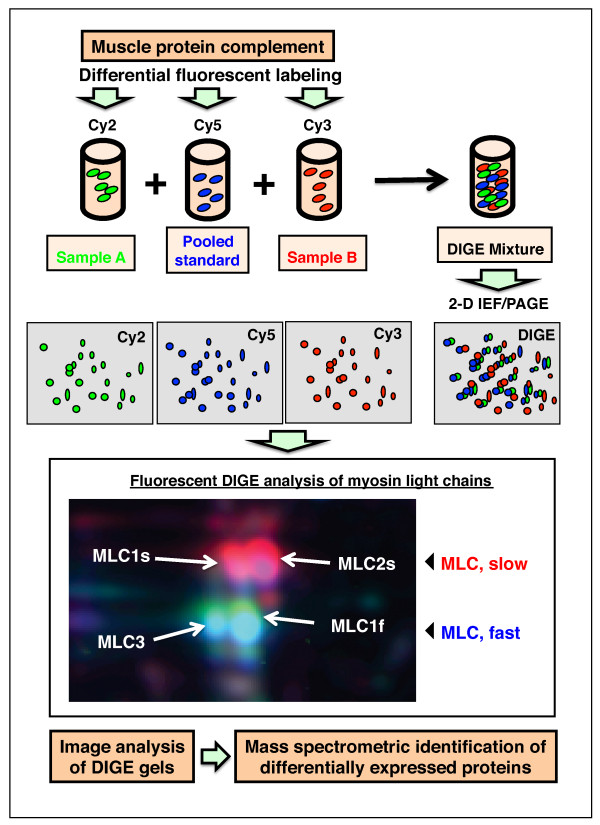
**Overview of the fluorescence difference in-gel electrophoretic (DIGE) method**. Shown is a diagram of the differential labeling of muscle specimens with the fluorescent CyDyes Cy2, Cy3 and Cy5, as well as an example of a DIGE analysis of myosin light chain isoforms during fast-to-slow transitions of electrostimulated skeletal muscle.

The DIGE method has also captured a lot of attention in the field of skeletal muscle proteomics over the past few years [55-57,59,63-66]. If the experimental design of fluorescent studies maximises the sensitivity for detecting changes in protein expression levels and takes into account statistical variations in dye binding, labelling artefacts of soluble protein species can be kept to a minimum [105-107]. Therefore, reverse DIGE labelling controls are not routinely employed. Dye-to-dye variability is usually minimal, and the findings from expression analyses with different dye combinations do not differ to a large extent. Analytical DIGE systems can be employed with two-dye or three-dye systems, depending on specific applications. Advanced DIGE, using an internal pooled standard, is a highly accurate quantitative method that enables multiple protein samples to be separated on the same two-dimensional gel. For example, a recent study conducted at our laboratory of the effects of experimental exon skipping on the dystrophic diaphragm from the x-linked muscular dystrophy (mdx) model of Duchenne muscular dystrophy combined the proteomic profiling of normal versus mdx versus treated mdx specimens [65]. In one set of six DIGE gels, 12 individual biological replicates of muscle samples were differentially labelled with different CyDyes Fluors (GE Healthcare Amersham Biosciences UK, Little Chalfont, Buckinghamshire, UK) for direct comparison on two-dimensional gels. A pool of all protein samples was also prepared and labelled with another CyDye to be employed as a standard on all gels. A pooled standard greatly aids image matching and cross-gel statistical analysis. Ideally, samples are evenly distributed between both CyDye fluors and analytical gels [65]. Figure 3 outlines the use of differential dye labelling for the analysis of two different muscle specimens. To illustrate the sensitivity of the fluorescent method, an enlarged DIGE image of distinct changes in the isoform expression pattern of myosin light chain during fast-to-slow muscle transitions is shown. Our proteomic profiling of fast muscle following chronic low-frequency stimulation has been studied by using DIGE analysis, and it clearly revealed a switch to slower isoforms of myosin light and heavy chains, as well as increased levels of oxidative enzymes [63].

### Subcellular proteomics of skeletal muscle

**Figure 4:**
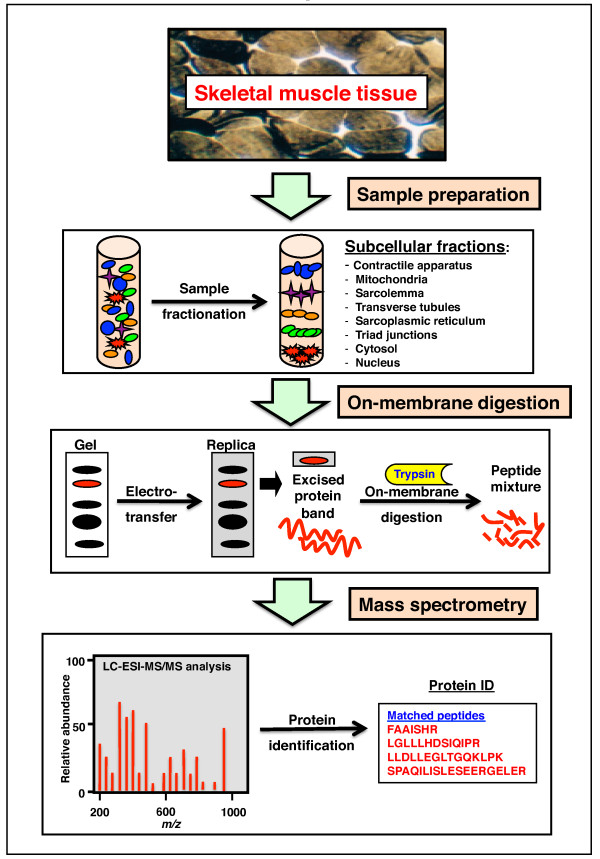
**On-membrane digestion method for muscle proteomics**. The flowchart outlines the application of on-membrane digestion for the mass spectrometric identification of membrane proteins and high molecular mass proteins from skeletal muscle tissues.

Large-scale gel electrophoretic and liquid chromatographic methods have limitations with respect to analysing highly complex protein mixtures and protein populations with an extensive dynamic expression range. Thus, to reduce sample complexity, organelle proteomic studies have been initiated that focus on the protein complement of distinct subcellular fractions. Organelle proteomic approaches include sample prefractionation and the use of narrow pH ranges for the isoelectric focusing of low copy number proteins in two-dimensional gels as well as in one-dimensional gels for studying hydrophobic and high molecular mass proteins [108-110]. Reference maps of subcellular fractions from skeletal muscle include microsomes, sarcolemma, cytosol, contractile apparatus and mitochondria [111-120]. Since mitochondria are involved in various diseases and cellular aging, many subcellular proteomic studies have focused on this crucial organelle [121-123]. Proteomic profiling of the mitochondria-enriched fraction from senescent rat muscle has revealed a shift to more aerobic oxidative metabolism in a slower-twitching fibre population during age-related muscle degeneration [118]. This organelle proteomic study has identified many new potential biomarkers of sarcopenia of old age [14]. Maughan *et al*. [111] showed that the 10 glycolytic enzymes represent the most abundant proteins in the diffusible fraction of the rabbit skeletal muscle proteome. This makes the key metabolic proteins that mediate the core glycolytic pathway ideal candidates to be studied by using mass spectrometry-based proteomics [95]. In contrast, organelle-associated proteins are usually more difficult to identify and characterise. In this respect, Figure 4 shows a novel approach for studying subcellular fractions by on-membrane digestion of electrophoretically transferred proteins.

An inefficient trypsination of certain target proteins often hampers in-gel digestion procedures. To address this technical problem in the proteomic identification of proteins, on-membrane digestion has been developed [124-127]. The advantage of on-membrane digestion is superior protein sequence coverage [124]. In addition, on-membrane digestion is more efficient and faster compared with conventional in-gel digestion methods [127]. This technical fact reduces complications due to trypsin autolysis and makes the on-membrane digestion technique especially suitable for the mass spectrometric identification of low copy number proteins and large hydrophobic proteins. This technique has recently been applied to the biochemical analysis of the large-membrane cytoskeletal protein dystrophin from rabbit skeletal muscle [120]. Standard two-dimensional gels are usually unable to separate muscle proteins with a molecular mass greater than 200 kDa [56]. In contrast, one-dimensional gradient gels are capable of presenting very large membrane proteins up to 2,000 kDa [128]. The mass spectrometric analysis of muscle membranes using one-dimensional gels identified membrane-associated muscle proteins as well as the Dp427 isoform of dystrophin, including the dihydropyridine receptor of the transverse tubules and the 565-kDa ryanodine receptor Ca^2+^-release channel of the junctional sarcoplasmic reticulum [120]. On-membrane digestion was demonstrated to be highly suitable for studying high molecular mass proteins that would otherwise not be properly recognised by gel electrophoresis-based proteomic studies. Muscle proteins adsorbed onto nitrocellulose sheets appear to be more accessible to proteases, increasing digestion efficiency [120]. This makes this novel technique the method of choice for studying large membrane proteins.

**Figure 5:**
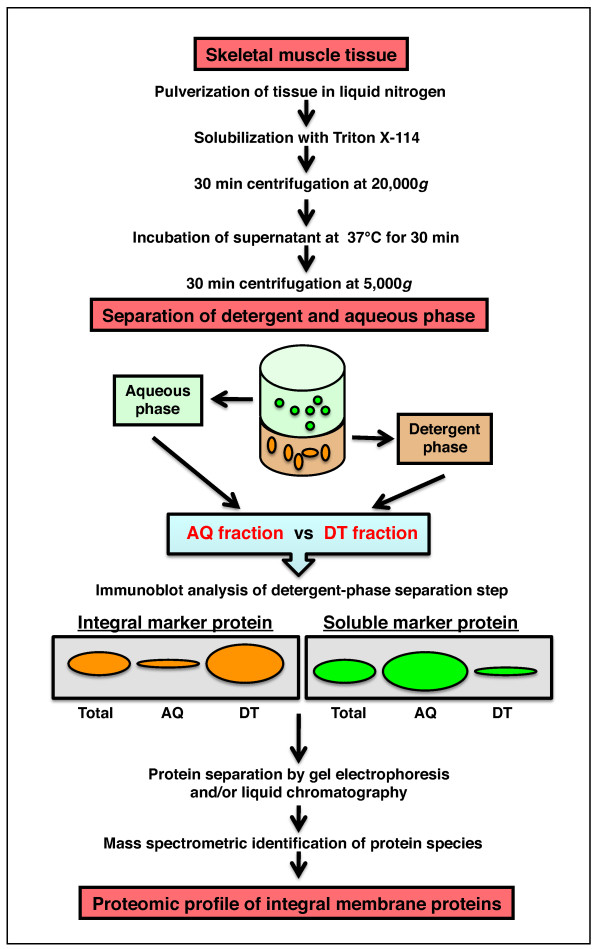
**Detergent phase extraction method for muscle proteomics**. The flowchart outlines the application of detergent phase extraction for the mass spectrometric identification of membrane-associated proteins from skeletal muscle tissues. The separation of protein species into an aqueous (AQ) fraction versus a detergent (DT) fraction can be conveniently verified by immunoblotting with antibodies to soluble versus integral markers.

Since membrane proteins play a crucial role in cellular signaling, regulation, homeostasis and metabolism, the large-scale identification and characterisation of peripheral and integral membrane proteomics is an important aspect of modern proteomics [129-131]. Recently, detergent phase extraction was applied to the proteomic analysis of the membrane-associated fraction from skeletal muscle [132]. This method is based on the principle of temperature-dependent phase extraction with Triton X-114. Since phase separation with this nonionic detergent occurs at temperatures above 22°C, skeletal muscle protein fractionation was carried out at 37°C [132]. As outlined in the flowchart in Figure 5, the separation step resulted in an aqueous phase enriched in hydrophilic proteins and a detergent phase with predominantly hydrophobic proteins. However, phase transition approaches always result in a certain degree of cross-contamination between soluble and membrane-associated proteins. The partitioning and cross-contamination of hydrophobic versus hydrophilic protein populations can be conveniently monitored by immunoblotting (Figure 5).

### Emerging technologies for muscle proteomics

Mass spectrometry-based proteomics is a fast-moving field with a variety of emerging technologies that have not yet been fully exploited in the field of skeletal muscle proteomics. Although several studies on muscle cells have included quantification methods involving the incorporation of stable isotopes through metabolic or chemical labelling [53,61,82,133-135], as well as through label-free shotgun proteomics [50,51,85,136,137], future applications of a variety of advanced gel-free techniques promise an even greater impact of proteomics on muscle biology and biomarker discovery. The advantages and technical challenges of label-free proteomics versus the use of stable isotopes for metabolic or chemical labelling have been extensively outlined in several excellent reviews [4,138-140]. Numerous stable isotope-labelling techniques have been employed in quantitative shotgun proteomics, including isobaric tags for relative and absolute quantification, or iTRAQ; isotope-coded affinity tag, or ICAT; and stable isotope labelling by amino acids in cell culture, or SILAC. In skeletal muscle proteomics, the SILAC method was successfully applied to the quantitative profiling of differential protein expression between myoblasts and myotubes [53] and the identification of proteins that interact with the glucose transporter GLUT4 in an insulin-regulated manner [133]. The ICAT labelling approach has been used for the proteomic profiling of the cytosolic fraction from atrophying mouse tibialis anterior muscle [135]. The iTRAQ technique was employed for the analysis of changes in the protein complement of human vastus lateralis muscle in response to interval exercise training [61], the cataloguing of the skeletal muscle proteome from pigs [82] and the identification of carbonylated proteins from rat muscle mitochondria [134].

In future proteomic shotgun experiments, protein populations from skeletal muscle tissues could be profiled by label-free quantification approaches such as peptide spectral counts. The term *spectral counts *refers to the number of mass spectrometry (MS)/MS identifications per protein species. Since abundant peptide species are detectable across a wide period of retention time and are therefore repeatedly sampled, a linear relationship exists between protein concentration and spectral counts [141]. This makes identification results from MS/MS spectra an attractive option for quantitative shotgun proteomics. With respect to the validation of protein biomarkers, stable isotope dilution (SID)-MS might be a useful addition to the analytical repertoire of muscle proteomics. This technique is especially useful for the characterisation of low-abundance biomarkers. Multiple reaction monitoring (MRM) coupled with SID-MS represents an ideal method for the quantitative measurement of low-abundance biomarkers [142]. In SID-MRM-MS investigations, the quantification of a biomarker protein of interest is achieved by selecting signature peptides of the digested target protein [143]. Unique signature peptides are employed as quantitative substitutes of the marker protein. The concentration of the biomarker protein can be determined by comparing the signals from the exogenous SID-labelled signature peptide and the endogenous unlabelled peptide population [144].

To tackle the technical challenges associated with analysing membrane proteins, an exciting universal sample preparation method has been developed: filter-aided sample preparation (FASP). On the basis of the fact that membrane proteins can be depleted of detergent by gel filtration in 8 M urea [145], Wisniewski *et al*. [146] established a proteomic sample preparation technique based on the complete solubilisation of a protein complement in 4% sodium dodecyl sulphate and subsequent exchange of detergent by urea on spin filter devices. The FASP method depletes the suspension of low molecular mass components in urea-containing buffer, digests a representative cohort of proteins including hydrophobic elements and elutes pure peptide mixtures [146]. Thus, FASP is an ideal tool to be applied to the proteomic identification and characterisation of high molecular mass membrane proteins from skeletal muscle, because peptide mixtures eluted after digestion on the spin filter were highly suitable for mass spectrometric analysis [146]. Since a large number of hydrophobic proteins are involved in physiological adaptations and pathological changes in contractile fibres, this new universal sample preparation tool promises to be extremely useful for future proteomic investigations of muscle organelles.

## Conclusions

Over the past few years, skeletal muscle proteomics has successfully catalogued the majority of abundant and soluble fibre-associated proteins. The refined proteomic analysis of isoform expression patterns and biochemical studies of dynamic posttranslational modifications has identified thousands of distinct muscle protein species. Myogenesis, muscle maturation, muscle transformation and aging-related muscle wasting have been intensively investigated by using proteomic methods and has resulted in the establishment of a comprehensive biomarker signature for major physiological adaptation processes in contractile tissues. The proteomic characterisation of common neuromuscular disorders has revealed novel disease-specific marker proteins of disuse atrophy, muscular dystrophy, obesity, type 2 diabetes, sepsis, hypokalemic myopathy, inclusion body myositis and reducing body myopathy. Thus, MS-based proteomics has decisively improved our general understanding of physiological and pathophysiological mechanisms in muscle tissues. New biomarker candidates can now be used for improving diagnostic methods, the identification of novel therapeutic targets, better comprehension of the molecular pathogenesis of muscular disorders, improved monitoring of disease progression and the judging of potential side effects of experimental drugs. Importantly, if the proteomic workflow could be successfully miniaturised, then single-cell proteomics of different fibre populations would lead to more comprehensive coverage of the skeletal muscle proteome. This depends on technical developments in the field of MS [20-22]. In the future, organelle and membrane proteomics will probably play a more prominent role in muscle biochemistry to study less abundant and more hydrophobic proteins. Filter-aided sample preparation and on-membrane digestion may be preferred for the proteomic analysis of high molecular mass membrane proteins. Once the majority of large and integral muscle proteins have been catalogued by proteomics, it will be crucial to correlate these findings with genomic, transcriptomic and metabolomic databanks [147] and establish the global relationship of biomolecules in striated voluntary muscle tissues.

## Competing interests

The author declares that they have no competing interests.
